# Prognostic value of CD34 expression status in patients with myxofibrosarcomas and undifferentiated pleomorphic sarcomas

**DOI:** 10.1038/s41598-021-94834-w

**Published:** 2021-07-29

**Authors:** Yoshiya Sugiura, Rikuo Machinami, Seiichi Matsumoto, Hiroaki Kanda, Keisuke Ae, Yutaka Takazawa, Kengo Takeuchi

**Affiliations:** 1 Division of Pathology, The Cancer Institute, Japanese Foundation for Cancer Research, 3-8-31, Ariake, Koto, Tokyo, 135-8550 Japan; 2 Department of Pathology, Toho University Medical Center-Sakura Hospital, Sakura, Japan; 3 Department of Pathology, Kawakita General Hospital, Tokyo, Japan; 4 Department of Orthopedic Oncology, Cancer Institute Hospital of Japanese Foundation for Cancer Research, Tokyo, Japan; 5 Department of Pathology, Saitama Cancer Center, Kita-Adachi, Japan; 6 Department of Pathology, Toranomon Hospital, Tokyo, Japan

**Keywords:** Medical research, Oncology

## Abstract

It is controversial whether patients with myxofibrosarcomas (MFSs) have better prognoses than those with undifferentiated pleomorphic sarcomas (UPSs). No useful prognostic factors have been established to date. We therefore aimed to evaluate the prognostic value of CD34 expression status in 192 patients with MFSs and UPSs. Using the log-rank test, we showed that patients with MFSs had a significantly better overall survival than did those with UPSs when defining the former as having a > 10% myxoid component (*p* = 0.03), but not when defining it as having a > 50% myxoid component (*p* = 0.1). Under the definition of MFSs as > 10% myxoid component, the log-rank test revealed that the diagnosis of the UPS and the CD34 loss (*p* < 0.001) were significant adverse predictors of overall survival. As per the Cox model, the CD34 loss remained an independent prognostic factor (hazard ratio = 3.327; 95% confidence interval 1.334–8.295), while the diagnosis of the UPS was a nonsignificant confounding factor (hazard ratio = 1.084; 95% confidence interval 0.679–1.727). In conclusion, CD34 expression status is a useful prognostic factor in patients with MFS and UPS, and it should be incorporated into grading systems that are used to predict outcomes.

## Introduction

Approximately 20–25% of soft tissue sarcomas are undifferentiated myxoid or pleomorphic tumors that have been referred to as malignant fibrous histiocytomas (MFHs) for more than 20 years. MFHs were categorized into two types, myxofibrosarcomas (MFSs) and undifferentiated pleomorphic sarcomas (UPSs), according to the recent World Health Organization classification^1^. Clinically, MFSs occur predominantly in the superficial regions of the extremities of elderly people; the tumor is histologically similar to the myxoid MFH characterized by an undifferentiated and myxoid appearance^1–4^, while the UPS is nearly identical to the non-myxoid MFH.

In the current Fédération Nationale des Centres de Lutte Contre le Cancer (FNCLCC) tumor-grading system, patients with MFSs are considered to have better prognoses than those with UPSs; the tumor differentiation scores of MFSs and UPSs are two and three, respectively. However, it is difficult to distinguish MFSs from UPSs because (1) the cutoff point for the extent of myxoid component that would distinguish MFSs from UPSs has not been established and varies widely from 5 to 50%^3, 5–7^, and (2) no specific genetic abnormalities have been found in either tumor type^8–12^. The Cancer Genome Atlas Research Network considers MFSs and UPSs to be on the spectra of a single disease^12^. Thus, the diagnosis of MFSs or UPSs is not always reliable and does not correlate with a consistent prognosis. Therefore, we aimed to identify prognostic factors in patients with MFSs and UPSs.

It has been reported that some patients with MFSs and UPSs are positive for CD34^13^, which is a transmembrane glycoprotein expressed in the fibroblasts as well as in the hematopoietic, endothelial, muscle satellite, and hair follicle cells^14^. Previous investigators have proposed that CD34 loss is associated with the malignant progression in several mesenchymal tumors such as dermatofibrosarcoma protuberans^15, 16^, solitary fibrous tumors^17, 18^, and phyllodes tumors^19–21^. We hypothesized that CD34 loss is an adverse prognostic factor for patients with MFSs and UPSs, and we conducted survival analyses to investigate this effect.

## Results

### Patient selection

Between 1979 and 2016, there were 261 patients diagnosed with a MFS, UPS, or MFH who underwent wide resection of their primary tumors. Two patients whose follow-up was censored within 200 days were excluded, as were 56 who lacked essential clinical information or for whom additional immunohistochemistry could not be performed. We then conducted immunohistochemistry on samples from all patients and excluded 11 that exhibited a specific line of differentiation, including five with leiomyosarcoma, three with dedifferentiated liposarcoma, two with pleomorphic rhabdomyosarcoma, and one with epithelioid sarcoma. Ultimately, 192 patients were included for the analysis of overall survival. The overall survival observation period ranged from 22 days to 35 years (median: 9.5 years). When calculating local recurrence-free survival and distant-metastasis-free survival, we excluded an additional 21 patients with metastatic lesions or nodal involvement at the time of the initial surgery. The disease-free survival observation periods ranged from 203 to 9381 days (median: 1163 days).

### Histological findings

**Figure 1:**
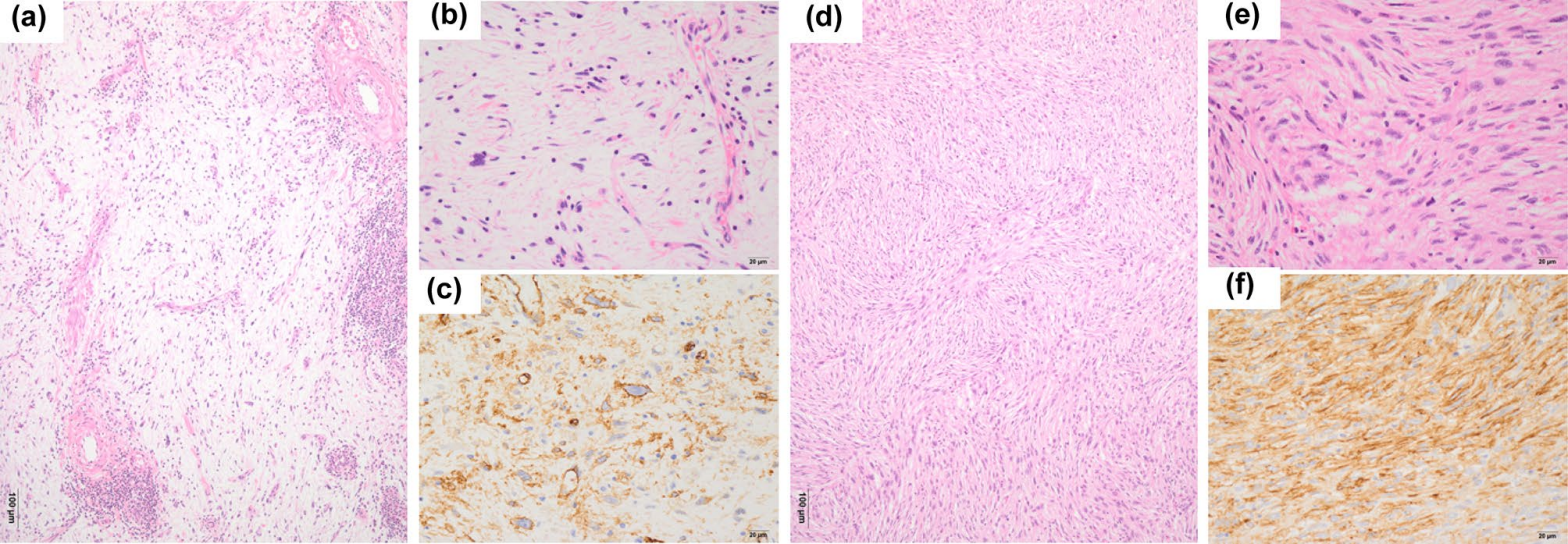
Histology and CD34-immunostaining of myxofibrosarcoma (MFS) and undifferentiated pleomorphic sarcoma (UPS). (**a** and **b**) MFS: Pleomorphic sarcoma with myxoid stroma (hematoxylin and eosin stain; original magnification, ×40 in A and ×400 in B. (**c**) MFS: The cytoplasm and cell membrane are strongly positive for CD34 (immunostaining, original magnification, ×400). (**d** and **e**) UPS: Pleomorphic sarcoma with non-myxoid stroma (hematoxylin and eosin stain; original magnification, ×40 in **d** and ×400 in **e**). (**f**) UPS: The cytoplasm and cell membrane are strongly positive for CD34 (immunostaining, original magnification, ×400).

Fifty-nine and 93 of the 192 samples were diagnosed as MFSs according to Weiss and Enzinger’s criteria and to Mentzel et al.’s criteria, respectively. Seven cases mimicked myxoma but focally exhibited pleomorphism. Eleven cases were rich in osteoclast-type giant cells. Six patients showed severe inflammatory changes, while reactive bone or chondroid tissue was also found in six patients. Of the 192 patients, 32 patients were positive for CD34. Tumor cell CD34 staining was membranous and/or cytoplasmic (Fig. 1). Among the 32 patients, 30 showed diffuse CD34-staining (on 50% or more tumor cells), while two patients showed focal CD34-staining (about 15% and 5% tumor cells); however, there were strong membranous and/or cytoplasmic staining on the tumor cell nests, and we considered them as focal CD34-positive cases.

### Characteristics of patients diagnosed with MFS versus UPS

**Figure 2:**
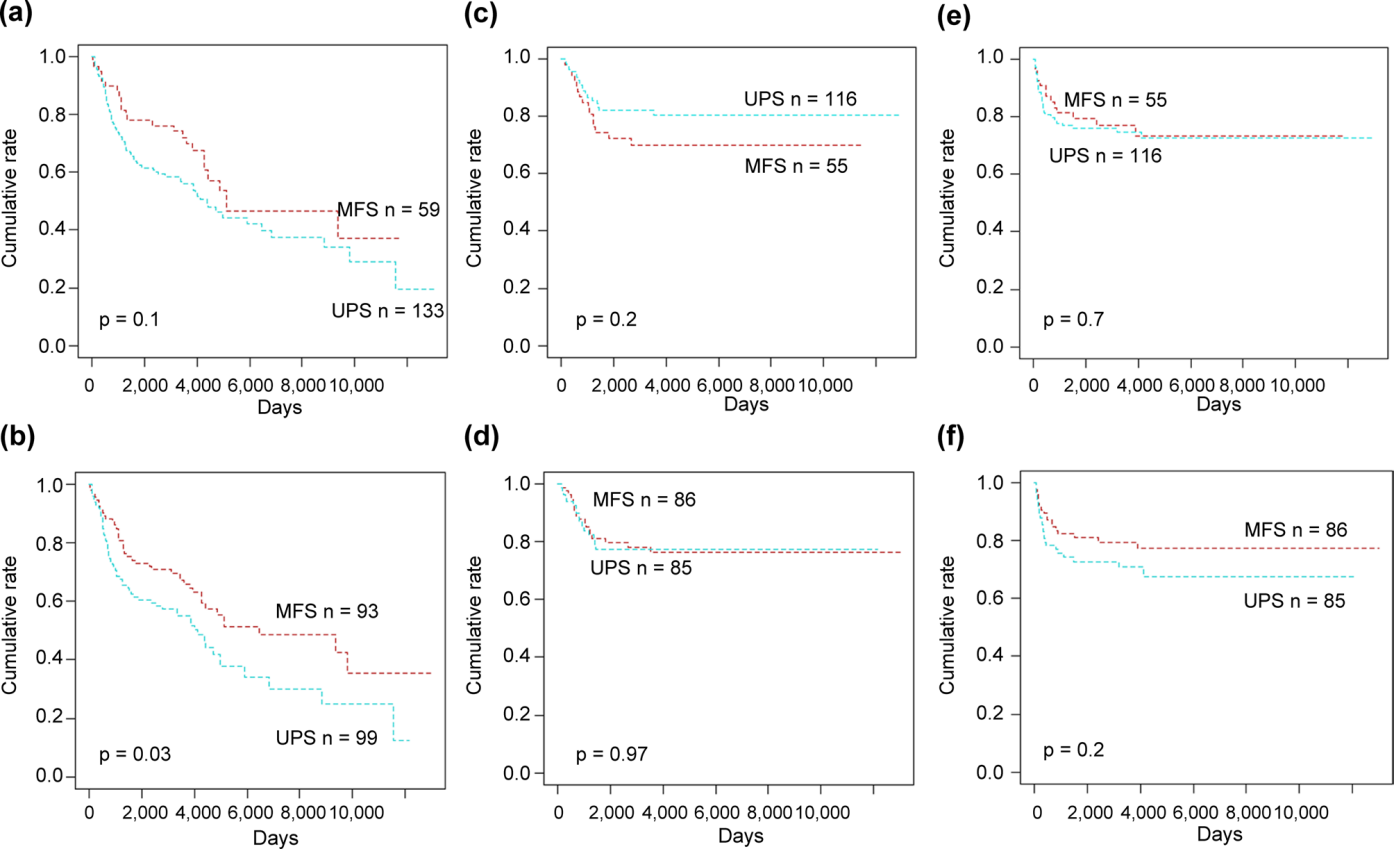
Survival curves of patients with myxofibrosarcoma (MFS) and undifferentiated pleomorphic sarcoma (UPS). When MFS was defined as a myxoid component of > 50%, there was no significant difference in overall survival between patients with MFS and those with UPS (**a**). On the other hand, patients with MFS showed significantly better overall survival than did those with UPS when MFS was defined as having a myxoid component > 10% (**b**). Local recurrence-free survival didn’t show significant differences between patients with the two types of diseases under the both definition of MFS (**c**: myxoid component of > 50%; **d**: > 10%). Distant metastasis-free survival also didn’t show significant differences between patients with the two types of diseases under the both definition of MFS (**e**: myxoid component of > 50%; **f**: > 10%).

Using Weiss and Enzinger’s criteria^5^, patients with MFSs (n = 59) tended to have a better overall survival than did those with UPSs (n = 133) (*p* = 0.1) (Fig. 2a), but the difference was not significant. On the other hand, using Mentzel et al.’s criteria^3^, patients with MFSs (n = 93) had significantly better overall survival than did those with UPSs (n = 99) (*p* = 0.03) (Fig. 2b). Thus, compared to Weiss and Enzinger’s criteria, Mentzel et al.’s criteria established a significant and more pronounced difference in overall survival between patients with MFSs and those with UPSs. Therefore, we used Mentzel et al.’s criteria to categorize all our patients into those with MFS versus UPS. Notably, neither local recurrence-free survival nor distant-metastasis-free survival was significantly affected by the diagnosis of MFS versus UPS under either criterion (Fig. 2c–f).

No significant differences were detected between the MFS and UPS patient groups with respect to median age (66 vs. 61 years; *p* = 0.19), mean tumor size (8.87 vs. 8.82, *p* = 0.52), or a number of other factors (Table 1).

**Table 1:** Patient characteristics.

Variables	Total (n = 192)	MFS (n = 93)	UPS (n = 99)	Comparison between MFS and UPS (*p* value)	CD34(+) (n = 32)	CD34(−) (n = 160)	Comparison between CD34(+) and (−) (*p* value)
**Age**							
≥ Median age	96	53	43	0.19 †	16	82	0.96†
< Median age	96	40	56		16	78	
**Size**							
≤ 5 cm	53	26	27	0.52 †	12	41	0.17†
> 5 cm	139	67	72		20	119	
**Gender**							
Male	103	48	55	0.58	20	77	0.14
Female	89	45	44		12	83	
**Site**							
Extremity	142	68	74	0.8	28	114	0.08
Trunk	50	25	25		4	46	
**Depth**							
Superficial	72	35	37	0.97	12	60	1
Deep	120	58	62		20	100	
**Nodal involvement**							
Negative	186	92	94	0.21	32	154	0.59
Positive	6	1	5		0	6	
**Distant metastasis**							
Negative	170	82	88	0.88	31	138	0.13
Positive	22	11	11		1	22	
**FNCLCC grade**							
Grade1	25	25	–	–	5	20	0.004*
Grade2	73	41	32		20	52	
Grade3	94	27	67		7	88	
**Surgical margin**							
Negative	155	70	85	0.06	23	132	0.25
Positive	37	23	14		9	28	
**Chemotherapy**							
Received	56	33	23	0.25	7	49	0.43
NOT received	136	66	70		25	111	

Bold values indicate *p* < 0.05

*p < 0.05.

For age and size, Mann–Whitney test was performed (†). For other factors, chi-square test was performed.

### Characteristics of patients who were CD34-positive versus CD34-negative

**Figure 3:**
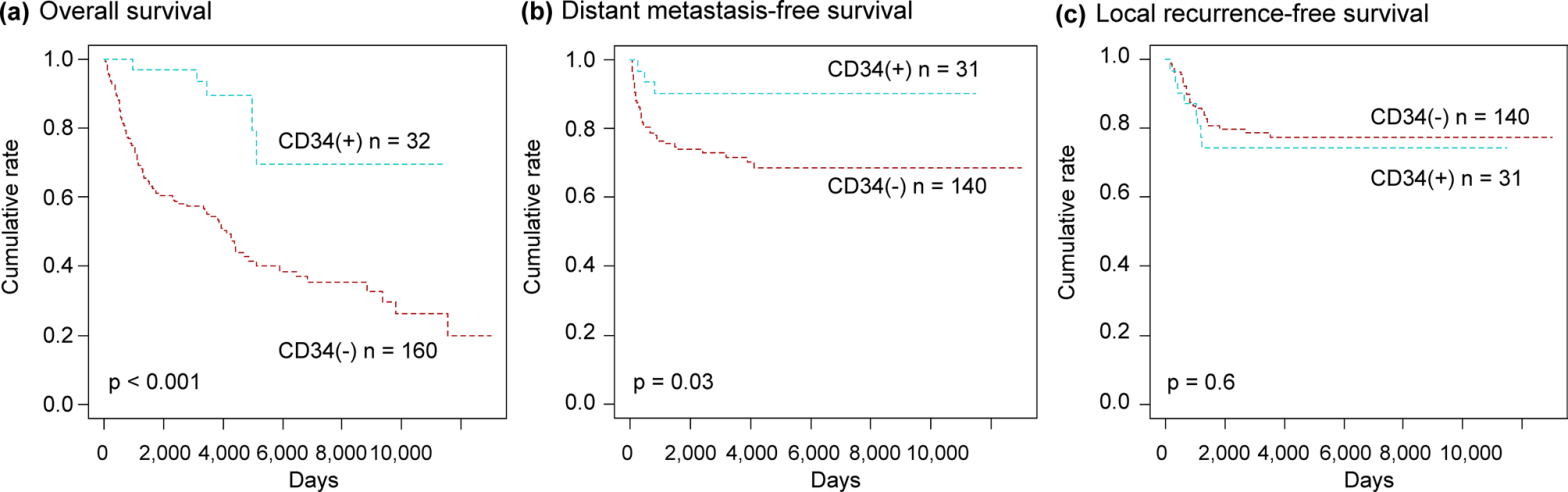
Survival curves of CD34-positive and CD34-negative patients with myxofibrosarcoma and undifferentiated pleomorphic sarcoma. Among all patients, those who were CD34-positive showed significantly better overall and distant metastasis-free survival (**a** and **b**), but not local recurrence-free survival (**c**), than those who were CD34-negative.

CD34-positive patients (n = 32) had a significantly better overall and distant metastasis-free survival than did CD34-negative patients (n = 160) (*p* < 0.001 and *p* = 0.027, respectively) (Fig. 3a, b), although local recurrence-free survival was not significantly influenced by their CD34 status (*p* = 0.6) (Fig. 3c).

No significant differences between CD34-positive and -negative patients were observed either in terms of median age (61 vs. 63 years, *p* = 0.96) or mean tumor size (9.39 vs. 8.61 cm, *p* = 0.88). In terms of histologic grade, the proportion of patients with grade 3 disease was significantly higher in the CD34-negative group (55%) than in the CD34-positive group (22%) (*p* = 0.004). However, no significant differences were found between the two groups with respect to the other factors (Table 1). A significantly higher proportion of patients with MFSs were CD34-positive than were patients with UPSs (23% [21 of 93] vs.11% [11 of 99], *p* = 0.03) (Table 2). The results of immunohistochemical analysis, other than CD34 and SMA in MFSs and UPSs, are also shown in Table 2 and Supplementary Table S1.

**Table 2:** Immunohistochemistry.

Antibody	Total (n = 192)		*p* value
	MFS (n = 93)	UPS (n = 99)	
**SMA**			
Positive	n = 28 (30%)	n = 44 (44%)	**0.04**
Diffusely (> 50%)	n = 19	n = 19	
Focally (< 50%)	n = 9	n = 25	
Negative	n = 65 (70%)	n = 55 (56%)	
**CD34**			
Positive	n = 21 (23%)	n = 11 (11%)	**0.03**
Diffusely (> 50%)	n = 20	n = 10	
Focally (< 50%)	n = 1	n = 1	
Negative	n = 72 (77%)	n = 88 (89%)	

Bold values indicate *p* < 0.05

### Univariate analyses of survival factors among all patients with MFS and UPS

In terms of overall survival, the diagnosis of UPSs as well as CD34 loss were significant adverse prognostic factors. A tumor size > 5 cm, deep tumor location, nodal involvement, distant metastasis, and high FNCLCC grade were also significant predictors of poorer overall survival (Figs. 2b, 3a, Table 3). The smooth muscle actin (SMA)-positive patients tended to have worse overall survival than did their SMA-negative counterparts, but the difference was not significant. Only a positive surgical margin was a significant predictor of poorer local recurrence-free survival (Table 3). The loss of CD34 was a significant predictor of poorer distant metastasis-free survival. Patients with tumors located in the trunk, tumor sizes > 5 cm, deeply located tumors, and high FNCLCC grade, also exhibited a significantly poorer distant metastasis-free survival (Table 3).

**Table 3:** Univariate analysis of clinico-pathologic factors with potential to affect overall survival (OS), local-recurrence free survival (LRFS), and distant-metastasis free survival (DMFS).

Clinicopathologic factors	OS (n = 192)	LRFS (n = 171)	DMFS (n = 171)
	*p* value	*p* value	*p* value
Age	0.12	0.7	0.38
Gender	0.26	0.4	0.33
Site	0.17	0.22	0.02*
Size	0.001*	0.580	0.02*
Depth	0.041*	0.052	0.19*
Nodal involvement	0.02*	NC	NC
Distant metastasis	< 0.001*	NC	NC
FNCLCC grade	< 0.001*	0.84	0.03*
Surgical margin	0.71	< 0.001*	0.98
CD34	< 0.001*	0.63	0.027 *
SMA	0.1	0.77	0.97
MFS vs UPS	0.04*	0.97	0.19

Bold values indicate *p* < 0.05

*p < 0.05.

*NC* not calculated.

### Multivariate analysis of factors independently affecting overall survival

In order of importance, CD34 loss (hazard ratio = 3.327), FNCLCC grade 3 (hazard ratio = 2.248), and the presence of a distant metastasis at the time of initial surgery (hazard ratio = 2.2) were independent prognostic factors (Table 4). The diagnosis of the UPS was only a confounding factor as it was not significant (*p* = 0.74). Nodal involvement was not significant (*p* = 0.51), probably because of insufficient nodal positive cases.

**Table 4:** Multivariate analysis of factors with potential to affect overall survival.

Variables	*p* value	Hazard ratio	95% CI
Size (≥ 5 cm vs. < 5 cm)	0.068	1.72	0.96–3.09
Depth (deep vs. superficial)	0.6	1.14	0.70–1.85
Histologic grade (G3 vs. G1)	0.002*	2.2	1.34–3.61
Nodal involvement (+ vs. −)	0.51	1.45	0.49–4.26
Distant metastasis (+ vs. −)	0.013*	2.25	1.19–4.26
CD34 (loss vs. expressing)	0.012*	3.33	1.33–8.30
The diagnosis of UPS	0.74	1.084	0.68–1.73

*p < 0.05.

### Effect of CD34 status on the overall survival of patients with MFS and UPS

**Figure 4:**
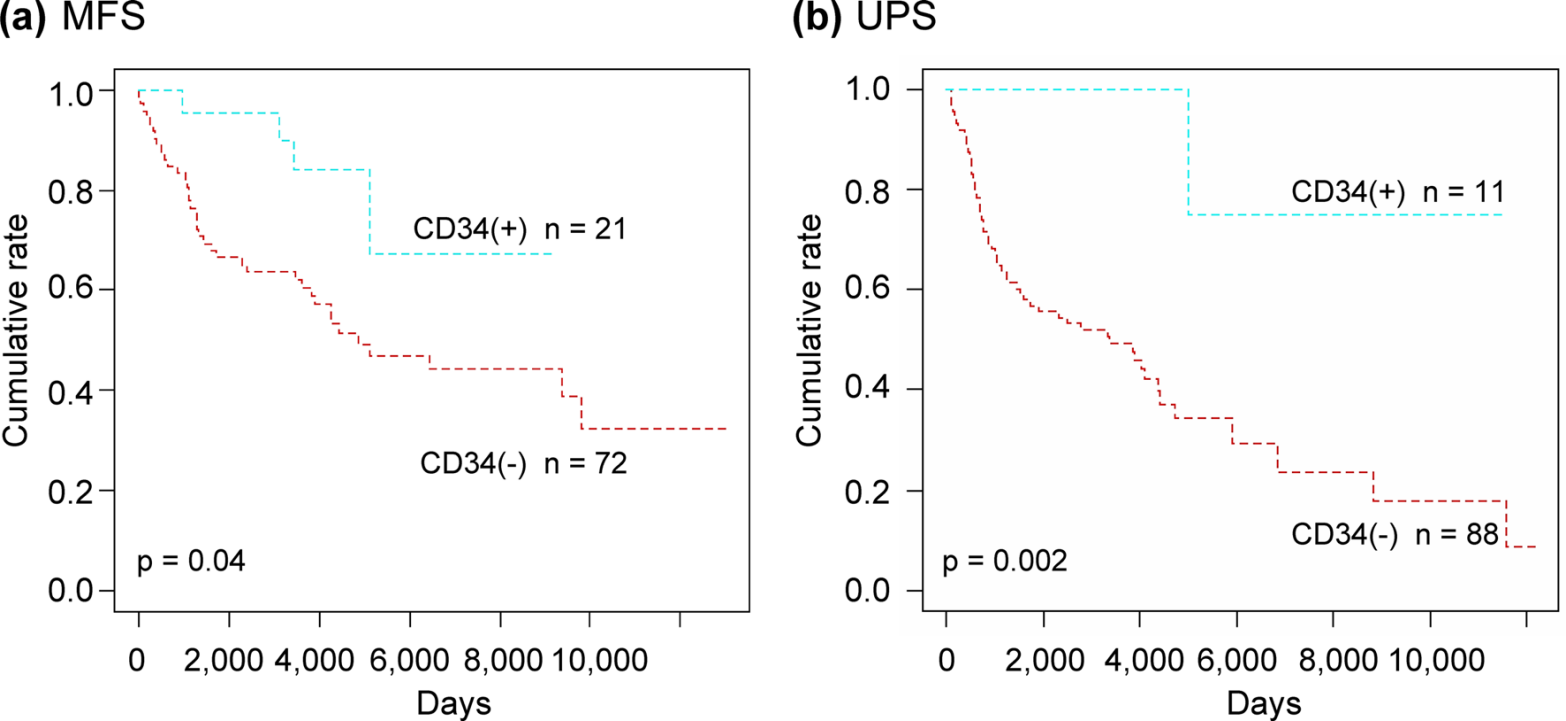
Overall survival of patients with myxofibrosarcoma (MFS) and undifferentiated pleomorphic sarcoma (UPS) according to CD34 status. Patients who were CD34-positive showed significantly better overall survival than did those who were CD34-negative whether they were diagnosed with MFS (**a**) or UPS (**b**).

CD34-positive patients had a significantly better overall survival than their CD34-negative counterparts, regardless of the diagnosis with either the MFS (*p* = 0.04) or UPS (*p* < 0.001) (Fig. 4a, b).

### Prognostic value of a UPS diagnosis in CD34-negative patinents

The diagnosis of the UPS was not a significant predictor of overall survival in patients who were CD34-positive (Supplementary Fig. S1A). In CD34-negative patients, however, the UPS was a significant adverse prognostic factor in both the univariate (Supplementary Fig. S1B, Supplementary Table S2) and multivariate analyses (Supplementary Table S3).

### Influence of CD34 status on the prognoses of patients who developed postoperative local recurrence or distant metastasis

Forty-three and 35 of 171 patients without preoperative metastatic lesions developed local recurrence and distant metastasis, respectively; eight patients developed both. Among patients with local recurrence and/or distant metastasis, those who were CD34-positive showed a significantly better overall survival than did those who were CD34-negative (*p* = 0.004, Supplementary Fig. S2).

### Effect of Chemotherapy on the overall survival of CD34-positive and CD34-negative patients

Compared with CD34-negative patients (*p* = 0.6), chemotherapy tended to more efficiently improve the overall survival of CD34-positive patients (*p* = 0.2), but this was not statistically significant (Supplementary Fig. S3A, S3B).

## Discussion

We showed that CD34 status was a useful predictor of overall survival in patients with MFSs and UPSs. The prognostic value of clinicopathologic factors such as MFS versus UPS and CD34 status were assessed in all our eligible patients. To date, it remains controversial whether patients with UPSs have worse prognoses than do those with MFSs^5, 22–24^. As per our univariate analysis, using Mentzel et al.’s criteria, the UPS was found to have a significantly worse overall survival than the MFS; moreover, CD34 loss was also found to be an adverse prognostic factor.

It was previously proposed that CD34 loss was associated with malignant progression in mesenchymal tumors such as dermatofibrosarcoma protuberans^15, 16^, solitary fibrous tumors^17, 18^, and phyllodes tumors^19–21^. However, the prognostic implication of CD34 loss had not been evaluated in patients with MFSs or UPSs. In our multivariate analysis, CD34 loss was found to be an independent predictor of overall survival, while the diagnosis of UPS was a confounding (i.e., nonsignificant) factor. We also found CD34 loss as an adverse prognostic factor regardless of whether the patients had MFSs or UPSs.

A significantly greater proportion of patients with MFSs exhibited CD34 positivity than did those with UPSs, which is consistent with a previous study^13^. The higher proportion of CD34 positivity among patients with MFS can contribute to their more favorable overall survival as observed on univariate analysis. However, this cannot be the sole reason that patients with MFS have better prognoses because, among the 160 CD34-negative patients, those with MFSs also had significantly better prognoses than did those with UPSs as per the results of the univariate and multivariate analyses. Hence, mechanisms other than CD34 immunopositivity may also be likely to render MFSs more indolent than UPSs. Further studies aimed at clarifying these mechanisms are necessary.

CD34-positive patients showed significantly better overall survival than did CD34-negative counterparts, which was likely because they developed significantly fewer distant metastases. However, even among patients who experienced postoperative local recurrence and/or distant metastasis, those who were CD34-positive still showed a significantly better overall survival than did those who were CD34-negative. While this finding is not easily explained, it is possible that CD34-positive patients are more sensitive to chemotherapy and/or radiotherapy; however, we were unable to investigate this hypothesis owing to the low number of patients who received such treatments (Supplementary Fig. S3A,S3 B).

We propose two mechanisms regarding the biological association between CD34 and the prognoses of patients with MFS and UPS. The first is associated with CD34-positive stromal fibroblastic/fibrocytic cells (CD34+ SFCs), which synthesize and remodel the extracellular matrix, thereby contributing to tissue repair, fibrosis, and tumor stroma formation. They also serve as mesenchymal progenitor cells that can differentiate into myofibroblasts, adipocytes, osteoblasts, or chondrocytes. CD34+ SFCs have been proposed as the progenitors of fibroblastic/myofibroblastic and lipomatous tumors^25^ since benign or low-grade malignant tumors of these types constantly express CD34^25, 26^ while their malignant counterparts frequently lack CD34 expression^15–21, 27^. It is possible that MFSs and UPSs initially develop as relatively well-differentiated fibroblastic/myofibroblastic sarcomas originating from CD34 + SFCs, but lose their CD34 expression while simultaneously gaining highly malignant features. However, it is also possible that CD34-positive tumors are derived from CD34+ SFCs while CD34-negative tumors have completely different origins; if such is the case, CD34-positive tumors could be considered a new tumor entity derived from CD34+ SFCs that are associated with better prognoses.

The second proposed mechanism is that CD34 suppresses tumor development, in which case our data could provide a basis for the development of novel therapeutic agents. The molecular mechanisms of CD34 should be clarified to better assess this possibility. This glycoprotein blocks differentiation and enhances the proliferation of stem or progenitor cells^14^. It also promotes the adhesion of L-selectin-expressing lymphocytes to the endothelium^14, 28^ and their recruitment of mast cells and eosinophils into intestinal and lung tissues^29, 30^. CD34 is also associated with intracellular signal transduction by interacting with the CT10 regulator of kinase-like protein^14, 30^. However, its molecular mechanism remains largely unknown, necessitating further studies.

Both the CD34 expression and the myxoid stroma percentage were interesting findings in our study. Patients with MFSs had a significantly better overall survival than did those with UPSs when the MFS patients were defined based on a > 10% myxoid component, but not on a > 50% myxoid component. If the sample size was larger, the definition of MFS based on a > 50% myxoid component would also have been relevant. However, despite the small sample size, the definition of MFS based on a > 10% myxoid component proved to be relevant. Therefore, we used a > 10% myxoid component to categorize all of our patients into those with MFS versus UPS. Both myxoid stroma and tumor cells can be related to the good prognosis of MFS. The reasons why MFSs had a better prognosis than UPSs could be explained by the existence of myxoid stroma as follows: (1) the number of cells in the MFSs was small compared with the tumor volume due to the existence of abundant myxoid stroma, and (2) myxoid stroma prevents the tumor cells from invading into the vessels. However, the existence of a myxoid stroma cannot fully explain the good prognoses of MFSs, because in our study, the cases with a small amount of myxoid stroma also had good prognoses. Probably, not only the myxoid stroma but also the tumor cells with the potential to produce myxoid stroma have factors related to the good prognosis, and it is necessary to clarify the molecular mechanism in future studies.

This study had some limitations. First because the diagnoses of MFSs and UPSs were based on the ruling out of other conditions, some cases could be rare variants of common sarcomas that are difficult to diagnose; we minimized the possibility of this to the best of our ability using immunohistochemistry. Second, there are two problems in the assessment of the CD34-positivity. One problem is whether the patients who revealed focal (< 50%) but evident staining should be considered as CD34-positive or not. We had two such cases, one died 4983 days after the initial wide resection, while the other is still alive 8837 days after the initial wide resection. Whether focal CD34-positivity affects the prognosis should be evaluated in a larger cohort. Another problem is the role of heterogeneity in CD34. It is a possible limitation to the use of CD34 as a prognostic marker. In a wide resection specimen, to perform immunohistochemistry on multiple tumor-rich sections may be effective. If CD34-positivity is assessed on a small biopsy specimen, the possibility of false-negative should be more carefully considered. Third, we did not assess the relationship between CD34 and each of periostin and SKIP2, which have recently been proposed as novel prognostic markers for both MFSs and UPSs^31, 32^.

In conclusion, CD34 status was a useful prognostic factor for patients with soft tissue sarcomas diagnosed as MFSs and UPSs. Therefore, we propose that CD34 status should be included in grading systems to predict the prognoses of patients with MFSs and UPSs.

## Methods

### Patient selection

Patients with sarcomas pathologically diagnosed as a MFS, UPS, or MFH and who underwent wide resection of their primary tumors between 1985 and 2016 were recorded in the database of the referral Cancer Institute Hospital, Japanese Foundation for Cancer Research. Of these patients, those whose follow-up was censored within 200 days were excluded, as were those lacking essential clinical information and/or those for whom additional immunohistochemistry could not be performed. We then performed additional immunohistochemistry to identify and exclude patients with specific types of differentiation such as adipocytic, smooth-muscle, skeletal-muscle, and epithelial tumors. This study was approved by the Institutional Review Board of the Cancer Institute Hospital (No. 2015-1158) of the Japanese Foundation for Cancer Research. All experiments were performed in accordance with relevant guidelines and regulations. Informed consent was obtained from all patients who were included in the study. If patients were under 18, informed consent was obtained from a parent and/or legal guardian.

### Tissue staining and immunohistochemistry

Tissues were processed following standard procedures. Formalin-fixed paraffin-embedded blocks were cut into 4 µm-thick sections and stained with hematoxylin and eosin.

Representative tumor-rich sections from the wide resection specimen of each sample were submerged in either a sodium citrate buffer or Tris–EDTA buffer for heat-induced epitope retrieval at 97 °C for 20 min. Immunostaining was performed using Leica Bond Polymer Refine Detection (Leica Biosystems, Wetzlar, Germany); the primary antibodies are described in Supplementary Table S1.

All slides were histologically reviewed by two pathologists (Y.S. and R.M.). All samples underwent immunostaining for desmin, SMA, S100, CD34, murine double minute type 2 (MDM2), and cytokeratin-AE1/AE3. Tumors were considered positive for desmin, S100, and cytokeratin-AE1/AE3 when 5% cells or greater were immunohistochemically stained, while the threshold for MDM2 and CDK4 was considered at 1% cells or greater on staining, based on the method of a previous study^33^. Tumors were considered positive for CD34 or SMA when the membrane and/or cytoplasm of overt sarcoma cells was immunohistochemically stained, regardless of the amount of the immunostaining of the cells. Diffusely-positive and focally-positive CD34 were defined as ≥ 50% and < 50% of positively-stained cells, respectively, based on the method used in a previous study^13^. Non-neoplastic fibroblasts/fibrocytes with CD34 and SMA-immunostaining and non-specific staining on fibrous stroma were carefully ruled out. We defined leiomyosarcoma as a sarcoma that was positive for both desmin and SMA; sarcomas that were positive for desmin but negative for SMA were subjected to further immunostaining for myogenin and Myo-D1 to test for rhabdomyosarcoma. For the CD34-positive samples, we conducted immunostaining for CD31 and ERG, STAT6, and integrase interactor-1 (INI-1) to test for angiosarcoma, solitary fibrous tumor, and epithelioid sarcoma, respectively. Furthermore, in CD34-positive patients, we excluded the possibility of dermatofibroma protuberans or myxo-inflammatory fibroblastic sarcoma by referring to the clinical information and histology. Samples exhibiting specific lines of differentiation were excluded.

### Statistical analysis

We divided the remaining cases (i.e., those without a specific differentiation type) into MFSs and UPSs according to the proportion of the myxoid component, for which two separate criteria have been proposed. Weiss and Enzinger defined the MFS as comprising a myxoid component of > 50%^5^, whereas Mentzel et al. defined it as having a myxoid component of > 10%^3^. Using a log-rank test we compared the differences in survival between the patients identified as having MFSs with identified as having UPSs in order to determine the threshold that corresponded to the greater difference in patient survival.

Next, we used the Kolmogorov–Smirnov test to assess the normality of continuous variables, and the Mann–Whitney test to assess the differences in age and tumor size of patients with MFSs and those with UPSs. Using the Chi-square test or Fisher’s exact test, the proportions of patients in each of the MFS and UPS groups were compared with respect to sex, tumor site, tumor depth, nodal involvement, distant metastasis, histologic grade, surgical margin at the time of the first wide resection, and whether they were receiving chemotherapy. We then categorized all patients as either CD34-positive or CD34-negative, and repeated the analytical tests. Using the Chi-square test, we also compared the proportions of CD34-positive patients with MFSs versus those with UPSs.

We identified for factors that significantly affected the prognoses of all patients with MFSs and UPSs by conducting survival analyses in terms of the following factors: MFSs vs. UPSs, immunopositivity for CD34 and SMA, age, tumor size, sex, tumor site, tumor depth, nodal involvement, distant metastasis, histologic grade, surgical margin at the time of the first wide resection of the primary tumor**,** and whether they were receiving chemotherapy. To evaluate the prognostic value of each factor, we calculated survival curves using the Kaplan–Meier method and compared them using the log-rank test for each factor. Survival was calculated from the date of wide resection of the primary tumor; the endpoints of overall survival, local recurrence-free survival, and distant-metastasis-free survival were the times of death, local recurrence, and distant metastasis, respectively. Data collection was censored on March 31, 2019.

The overall survival of all 192 patients with MFSs and UPSs was calculated; however, patients with metastatic lesions and nodal involvement were excluded when calculating local recurrence-free survival and distant-metastasis-free survival. Multivariate analyses were performed using the Cox proportional hazards model to simultaneously evaluate the effects of several factors on overall survival. Factors with *p* values < 0.05 in the univariate analyses were included in this model.

Next, we evaluated the prognostic significance of CD34 positivity in terms of overall survival separately, in patients with MFSs and UPSs using the log-rank test. The influences of MFS versus UPS diagnoses on overall survival, local recurrence-free survival, and distant metastasis-free survival were also examined individually in the CD34-positive and -negative patients using the log-rank test and Cox’s model. Finally, in patients with a postoperative local recurrence and/or distant metastasis, overall survival was compared between the CD34 positive and -negative patients using the log-rank test.

For all the statistical analyses in this study, the threshold for significance was a two-tailed *p* value of < 0.05. These analyses were conducted using SPSS version 19 (IBM Corp., Armonk, NY, USA) and R version 3.6.2 (R Foundation for Statistical Computing, Vienna, Austria).

The raw data used in this study are available from the corresponding author upon reasonable request.

## Supplementary Information

Supplementary Information.

## Data Availability

The datasets generated during and/or analysed during the current study are available from the corresponding author on reasonable request.
