# Supplementary material for: Prognostic value of CD34 expression status in patients with myxofibrosarcomas and undifferentiated pleomorphic sarcomas
Source: Sci Rep. 2021 Jul 29;11:15494. doi: 10.1038/s41598-021-94834-w (PMC8322140; doi:10.1038/s41598-021-94834-w)
Supplement: Supplementary file 1 — Supplementary Information. [file 41598_2021_94834_MOESM1_ESM.pdf]

Prognostic value of CD34 expression status in patients with myxofibrosarcomas and undifferentiated pleomorphic sarcomas

Yoshiya Sugiura, MD; Rikuo Machinami, MD, PhD; Seiichi Matsumoto, MD, PhD; Hiroaki Kanda, MD, PhD; Keisuke Ae, MD, PhD; Yutaka Takazawa, MD, PhD; Kengo Takeuchi, MD, PhD

Supplementary Figures

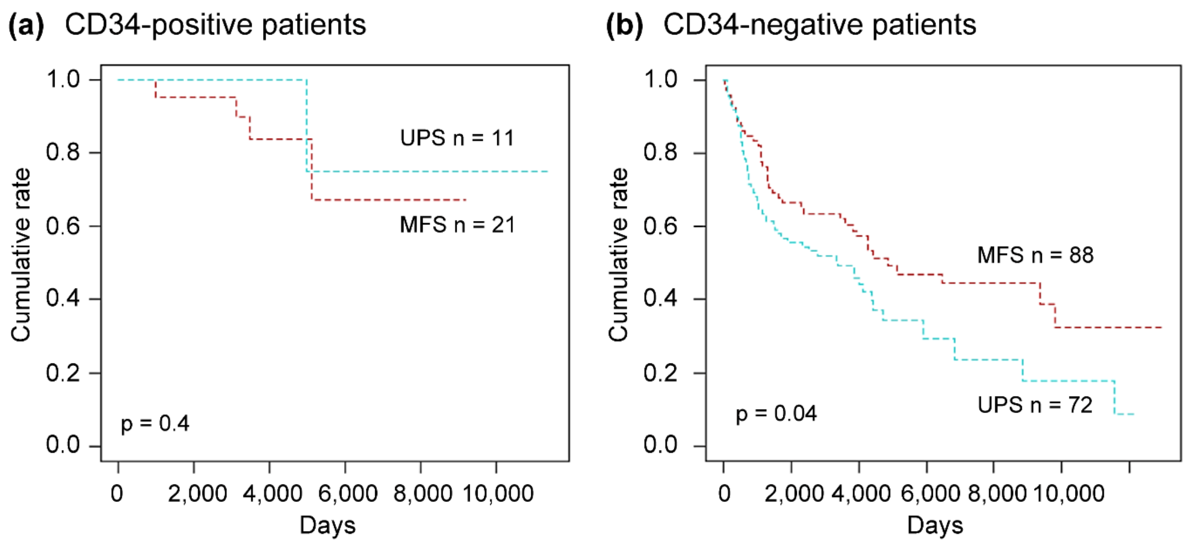

Supplementary Figure S1: Overall survival of patients who were CD34-positive versus CD34-negative according to disease histology.

Among patients who were CD34-positive, no significant difference was observed between those diagnosed with myxofibrosarcoma (MFS) versus undifferentiated pleomorphic sarcoma (UPS) (A). However, among patients who were CD34-negative, those with MFS showed significantly better overall survival than did those with UPS (B).

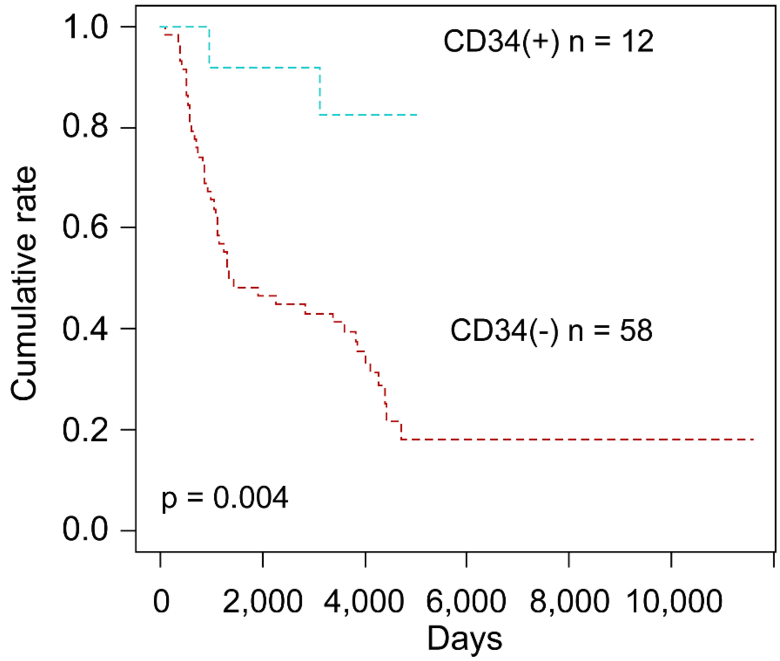

Supplementary Figure S2: Overall survival of patients with postoperative local recurrence and/or distant metastasis.

CD34-positive patients showed significantly better overall survival than their CD34-negative counterparts.

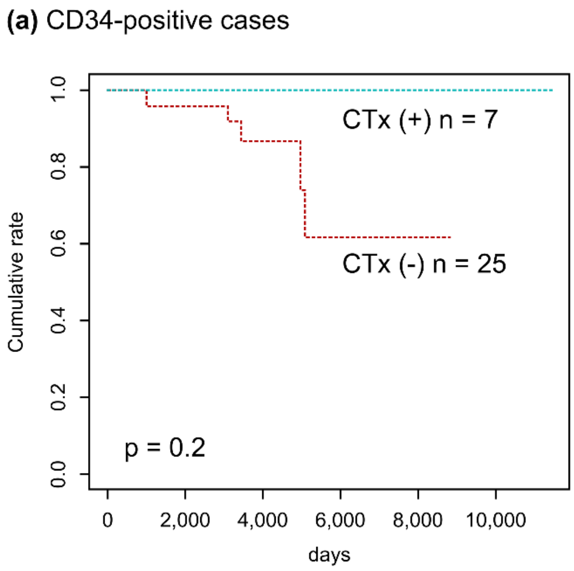

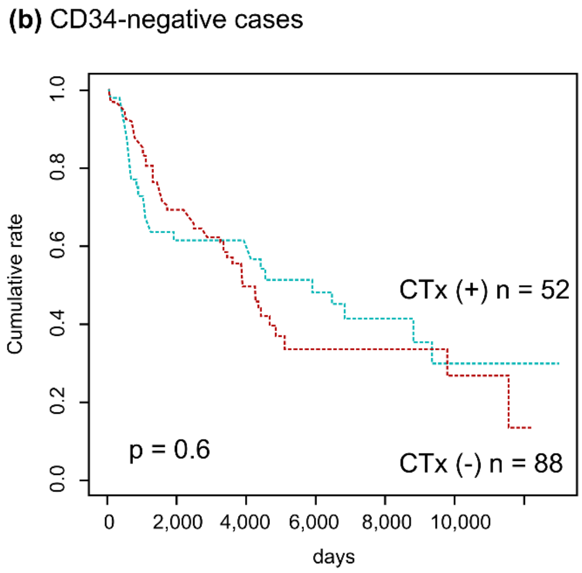

Supplementary Figure S3: Effect of chemotherapy on the survival of CD34-positive and CD34-negative patients

Compared to CD34-negative patients, in CD34-positive patients, chemotherapy tended to more efficiently improve the overall survival, but this was not statistically significant. CTx (+), chemotherapy was administered; CTx (-), chemotherapy was not administered

Supplementary Table S1. Immunohistochemistry

		Total (n=192)	
**Antibody**		*MFS (n = 93)*	*UPS (n = 99)*
Desmin			
Very focally positive (<10%)		n = 4	n = 10
Negative		n = 89	n = 89
S100			
Very focally positive (<10%)		n = 1	n = 6
Negative		n = 92	n = 93
CK-AE1/AE3			
Very focally positive (<10%)		n = 0	n = 1
Negative		n = 93	n = 98
MDM2			
Negative		n= 93	n = 99

Supplementary Table S2. Univariate analysis of clinicopathologic factors with potential to affect overall survival (OS), local-recurrence free survival (LRFS), and distant-metastasis free survival (DMFS) in CD34-negative cases

Clinicopathologic factors	***OS (n = 192)*** **p-value**	***LRFS (n = 171)*** **p-value**	***DMFS (n = 171)*** **p-value**
Age	0.19	0.7	0.4
Gender	0.06	0.4	0.3
Site	**0.04***	0.2	**0.03***
Size	**0.04***	0.6	**0.02***
Depth	**0.04***	**0.049***	0.2
Nodal involvement	**0.001***	NC	NC
Distant metastasis	**0.001***	NC	NC
FNCLCC grade	0.09	0.8	**0.04***
Surgical margin	0.71	**<0.001***	1
MFS vs UPS	**0.04***	1	0.2

* $p < 0.05$

NC: Not calculated

Supplementary Table S3. Multivariate analysis of factors with potential to affect overall survival in CD34-negative cases

Variables	p-value	Hazard ratio	95% CI
Diagnosis of UPS	**0.04***	1.574	1.022-2.425
Site (trunk vs extremity)	0.221	1.33	0.843-2.100
Depth (deep vs superficial)	0.334	1.261	0.788-2.017
Size (<5 cm vs ≥5 cm)	**0.034***	1.878	1.047-3.365
Nodal involvement (+ vs -)	0.444	1.535	0.513-4.597
Distant metastasis (+ vs -)	**0.042***	1.93	1.024-3.636

Supplementary Table S4. Primary antibodies

Antigen	Clone	Manufacturer	Dilution
Desmin	DE-R-11	Leica Biosystems, Newcastle, UK	1:100
SMA	1A4	Dako, Glostrup, Denmark	1:100
CD34	NU-4A1	Nichirei, Tokyo, Japan	1:5
S100	polyclonal	Leica Biosystems, Newcastle, UK	1:1000
CK-AE1/AE3	AE1 and AE3	Leica Biosystems, Newcastle, UK	1:200
MDM2	IF2	Invitrogen, Waltham, MA, USA	1:250
CDK4	polyclonal	Cell Signaling Technology, Danvers, MA, USA	1:400
Myogenin	F5D	Agilent, Santa Clara, CA, USA	1:500
Myo-D1	5.2F	Abcam, Tokyo, Japan	1:200
INI-1	25/BAF47	BD Transduction Labs, San Diego, CA, USA	1:500
CD31	JC70A	Agilent, Santa Clara, CA, USA	1:300
STAT6	polyclonal	Abcam, Tokyo, Japan	1:200
ERG	EP111	Abcam, Tokyo, Japan	1:100
